# Supplementary material for: Mechanistic insights into robust cardiac IKs potassium channel activation by aromatic polyunsaturated fatty acid analogues
Source: eLife. 2023 Jun 23;12:e85773. doi: 10.7554/eLife.85773 (PMC10328494; doi:10.7554/eLife.85773)
Supplement: Figure 1—source data 1. [file elife-85773-fig1-data1.docx]

| **Effects of tyrosine PUFA Analogs on IKs Channel** | | | | | | |
| --- | --- | --- | --- | --- | --- | --- |
| **Effects of NALT (n=4)** | | | | | | |
| Concentration | I/I_0_ Mean | I/I_0_ SEM | ΔV_0.5_ (mV) Mean | ΔV_0.5_ (mV) SEM | G_max_/G­_max0_ Mean | G_max_/G­_max0_ SEM |
| 0.2 μM | 0.94553 | 0.12581 | 3.575 | 0.38379 | 0.98609 | 0.03552 |
| 0.7 μM | 1.06501 | 0.1958 | 4.4 | 0.55827 | 1.13484 | 0.1989 |
| 2 μM | 1.9349 | 0.31466 | -4.95 | 2.75605 | 1.43115 | 0.25814 |
| 7 μM | 3.7006 | 0.66965 | -20.075 | 2.73538 | 1.53136 | 0.32306 |
| 20 μM | 5.14129 | 1.22612 | -56.125 | 3.58733 | 1.43185 | 0.31044 |
| **Effects of Lin-Tyrosine (n=4)** | | | | | | |
| Concentration | I/I_0_ Mean | I/I_0_ SEM | ΔV_0.5_ (mV) Mean | ΔV_0.5_ (mV) SEM | G_max_/G­_max0_ Mean | G_max_/G­_max0_ SEM |
| 0.2 μM | 1.22055 | 0.10582 | 0.21253 | 2.22787 | 1.227513 | 0.070988 |
| 0.7 μM | 2.29763 | 0.19488 | -8.4223 | 1.76905 | 1.441453 | 0.073856 |
| 2 μM | 4.89858 | 0.77774 | -23.405 | 1.6272 | 1.639097 | 0.15893 |
| 7 μM | 9.87097 | 2.04909 | -55.606 | 1.95738 | 1.564258 | 0.299473 |
| 20 μM | 12.7906 | 2.0734 | -74.426 | 4.13763 | 2.042834 | 0.589592 |
| **Effects of DHA-Tyrosine (n=3)** | | | | | | |
| Concentration | I/I_0_ Mean | I/I_0_ SEM | ΔV_0.5_ (mV) Mean | ΔV_0.5_ (mV) SEM | G_max_/G­_max0_ Mean | G_max_/G­_max0_ SEM |
| 0.2 μM | 1.32449 | 0.08717 | -1.6333 | 1.01368 | 1.220551 | 0.106905 |
| 0.7 μM | 2.00649 | 0.29274 | -10.51 | 2.6616 | 1.489147 | 0.122909 |
| 2 μM | 3.0083 | 0.52126 | -24.175 | 3.19479 | 1.728868 | 0.153379 |
| 7 μM | 4.74152 | 0.42339 | -50.563 | 4.2525 | 1.77601 | 0.24577 |
| 20 μM | 5.02345 | 0.92481 | -72.003 | 4.87459 | 1.46256 | 0.228565 |
| **Effects of Pin-Tyrosine (n=5)** | | | | | | |
| Concentration | I/I_0_ Mean | I/I_0_ SEM | ΔV_0.5_ (mV) Mean | ΔV_0.5_ (mV) SEM | G_max_/G­_max0_ Mean | G_max_/G­_max0_ SEM |
| 0.2 μM | 1.31377 | 0.06836 | -1.8812 | 2.94515 | 1.1645 | 0.127862 |
| 0.7 μM | 1.61785 | 0.143 | -5.3166 | 3.93447 | 1.287547 | 0.155134 |
| 2 μM | 2.57733 | 0.45741 | -15.125 | 5.46191 | 1.63353 | 0.295931 |
| 7 μM | 4.68209 | 0.65009 | -39.496 | 6.27025 | 1.833312 | 0.360226 |
| 20 μM | 5.78538 | 0.85995 | -60.524 | 5.76059 | 1.829174 | 0.436823 |
| **Effects of Lin-Glycine (n=3)** | | | | | | |
| Concentration | I/I_0_ Mean | I/I_0_ SEM | ΔV_0.5_ (mV) Mean | ΔV_0.5_ (mV) SEM | G_max_/G­_max0_ Mean | G_max_/G­_max0_ SEM |
| 0.2 μM | 1.09333 | 0.09025 | 1.25 | 0.40501 | 1.1 | 0.09 |
| 0.7 μM | 1.27333 | 0.13932 | -1.14667 | 0.9552 | 1.16333 | 0.07839 |
| 2 μM | 1.66667 | 0.1619 | -4.27667 | 2.24003 | 1.35 | 0.10017 |
| 7 μM | 3.84333 | 0.13544 | -17.11 | 1.32508 | 1.88667 | 0.14146 |
| 20 μM | 6.74667 | 1.05629 | -30.7633 | 5.43538 | 2.57333 | 0.48089 |
| Table containing source data for the application of the PUFA analogues NALT, Lin-Tyrosine, DHA-Tyrosine, Pin-Tyrosine and Lin-Glycine on the cardiac Kv7.1/KCNE1 at every concentration (0.2, 0.7, 2, 7, and 20 μM). | | | | | | |
