# Supplementary material for: Mechanistic insights into robust cardiac IKs potassium channel activation by aromatic polyunsaturated fatty acid analogues
Source: eLife. 2023 Jun 23;12:e85773. doi: 10.7554/eLife.85773 (PMC10328494; doi:10.7554/eLife.85773)
Supplement: Figure 2—source data 1. [file elife-85773-fig2-data1.docx]

| **Effects of tyrosine PUFA Analogs removing distal hydroxyl on IKs Channel** | | | | | | |
| --- | --- | --- | --- | --- | --- | --- |
| **Effects of NALT (n=4)** | | | | | | |
| Concentration | I/I_0_ Mean | I/I_0_ SEM | ΔV_0.5_ (mV) Mean | ΔV_0.5_ (mV) SEM | G_max_/G­_max0_ Mean | G_max_/G­_max0_ SEM |
| 0.2 μM | 0.94553 | 0.12581 | 3.575 | 0.38379 | 0.98609 | 0.03552 |
| 0.7 μM | 1.06501 | 0.1958 | 4.4 | 0.55827 | 1.13484 | 0.1989 |
| 2 μM | 1.9349 | 0.31466 | -4.95 | 2.75605 | 1.43115 | 0.25814 |
| 7 μM | 3.7006 | 0.66965 | -20.075 | 2.73538 | 1.53136 | 0.32306 |
| 20 μM | 5.14129 | 1.22612 | -56.125 | 3.58733 | 1.43185 | 0.31044 |
| **Effects of Lin-Tyrosine (n=4)** | | | | | | |
| Concentration | I/I_0_ Mean | I/I_0_ SEM | ΔV_0.5_ (mV) Mean | ΔV_0.5_ (mV) SEM | G_max_/G­_max0_ Mean | G_max_/G­_max0_ SEM |
| 0.2 μM | 1.22055 | 0.10582 | 0.21253 | 2.22787 | 1.227513 | 0.070988 |
| 0.7 μM | 2.29763 | 0.19488 | -8.4223 | 1.76905 | 1.441453 | 0.073856 |
| 2 μM | 4.89858 | 0.77774 | -23.405 | 1.6272 | 1.639097 | 0.15893 |
| 7 μM | 9.87097 | 2.04909 | -55.606 | 1.95738 | 1.564258 | 0.299473 |
| 20 μM | 12.7906 | 2.0734 | -74.426 | 4.13763 | 2.042834 | 0.589592 |
| **Effects of NAL-Phe (n=4)** | | | | | | |
| Concentration | I/I_0_ Mean | I/I_0_ SEM | ΔV_0.5_ (mV) Mean | ΔV_0.5_ (mV) SEM | G_max_/G­_max0_ Mean | G_max_/G­_max0_ SEM |
| 0.2 μM | 0.9599 | 0.04001 | 1.23454 | 0.35356 | 0.95975 | 0.03753 |
| 0.7 μM | 0.92202 | 0.06106 | 0.90146 | 0.20057 | 0.93473 | 0.04503 |
| 2 μM | 1.30025 | 0.19626 | -1.6853 | 1.21466 | 1.07294 | 0.07062 |
| 7 μM | 2.03309 | 0.48552 | -7.5297 | 3.02791 | 1.16998 | 0.13177 |
| 20 μM | 2.41388 | 0.50284 | -12.527 | 3.76753 | 1.15843 | 0.1482 |
| **Effects of Lin-Phe (n= 4)** | | | | | | |
| Concentration | I/I_0_ Mean | I/I_0_ SEM | ΔV_0.5_ (mV) Mean | ΔV_0.5_ (mV) SEM | G_max_/G­_max0_ Mean | G_max_/G­_max0_ SEM |
| 0.2 μM | 1.19706 | 0.13522 | -0.3449 | 0.7655 | 1.111297488 | 0.058825299 |
| 0.7 μM | 1.51092 | 0.27853 | -1.963 | 1.62673 | 1.209968371 | 0.102422216 |
| 2 μM | 1.81388 | 0.36027 | -4.4308 | 2.28685 | 1.234243176 | 0.083748917 |
| 7 μM | 2.38185 | 0.3655 | -7.534 | 3.35883 | 1.246369784 | 0.136421577 |
| 20 μM | 2.6371 | 0.31779 | -13.142 | 2.91986 | 1.238082757 | 0.098240949 |
| Table containing source data for the application of the PUFA analogues NALT, Lin-Tyrosine, NAL-Phe and Lin-Phe on the cardiac Kv7.1/KCNE1 at every concentration (0.2, 0.7, 2, 7, and 20 μM). | | | | | | |
