# Supplementary material for: Mechanistic insights into robust cardiac IKs potassium channel activation by aromatic polyunsaturated fatty acid analogues
Source: eLife. 2023 Jun 23;12:e85773. doi: 10.7554/eLife.85773 (PMC10328494; doi:10.7554/eLife.85773)
Supplement: Figure 3—source data 1. [file elife-85773-fig3-data1.docx]

| **Effects of electronegative phenylalanine PUFA Analogs on IKs Channel** | | | | | | |
| --- | --- | --- | --- | --- | --- | --- |
| **Effects of NALT (n=4)** | | | | | | |
| Concentration | I/I_0_ Mean | I/I_0_ SEM | ΔV_0.5_ (mV) Mean | ΔV_0.5_ (mV) SEM | G_max_/G­_max0_ Mean | G_max_/G­_max0_ SEM |
| 0.2 μM | 0.94553 | 0.12581 | 3.575 | 0.38379 | 0.98609 | 0.03552 |
| 0.7 μM | 1.06501 | 0.1958 | 4.4 | 0.55827 | 1.13484 | 0.1989 |
| 2 μM | 1.9349 | 0.31466 | -4.95 | 2.75605 | 1.43115 | 0.25814 |
| 7 μM | 3.7006 | 0.66965 | -20.075 | 2.73538 | 1.53136 | 0.32306 |
| 20 μM | 5.14129 | 1.22612 | -56.125 | 3.58733 | 1.43185 | 0.31044 |
| **Effects of NAL-Phe (n=4)** | | | | | | |
| Concentration | I/I_0_ Mean | I/I_0_ SEM | ΔV_0.5_ (mV) Mean | ΔV_0.5_ (mV) SEM | G_max_/G­_max0_ Mean | G_max_/G­_max0_ SEM |
| 0.2 μM | 0.9599 | 0.04001 | 1.23454 | 0.35356 | 0.95975 | 0.03753 |
| 0.7 μM | 0.92202 | 0.06106 | 0.90146 | 0.20057 | 0.93473 | 0.04503 |
| 2 μM | 1.30025 | 0.19626 | -1.6853 | 1.21466 | 1.07294 | 0.07062 |
| 7 μM | 2.03309 | 0.48552 | -7.5297 | 3.02791 | 1.16998 | 0.13177 |
| 20 μM | 2.41388 | 0.50284 | -12.527 | 3.76753 | 1.15843 | 0.1482 |
| **Effects of 4Br-NAL-Phe (n=3)** | | | | | | |
| Concentration | I/I_0_ Mean | I/I_0_ SEM | ΔV_0.5_ (mV) Mean | ΔV_0.5_ (mV) SEM | G_max_/G­_max0_ Mean | G_max_/G­_max0_ SEM |
| 0.2 μM | 1.10377 | 0.14642 | -1.1557 | 0.34856 | 1.01737 | 0.07177 |
| 0.7 μM | 2.17278 | 0.34428 | -6.0407 | 1.18471 | 1.55679 | 0.09312 |
| 2 μM | 3.32382 | 0.47586 | -12.482 | 1.95715 | 1.76235 | 0.10923 |
| 7 μM | 4.22364 | 0.33023 | -18.183 | 2.00472 | 1.87167 | 0.06235 |
| 20 μM | 4.59733 | 0.09241 | -22.816 | 2.04124 | 1.88249 | 0.10315 |
| **Effects of 4F-NAL-Phe (n=4)** | | | | | | |
| Concentration | I/I_0_ Mean | I/I_0_ SEM | ΔV_0.5_ (mV) Mean | ΔV_0.5_ (mV) SEM | G_max_/G­_max0_ Mean | G_max_/G­_max0_ SEM |
| 0.2 μM | 1.055 | 0.07805 | 0.5475 | 0.14522 | 1.0925 | 0.06343 |
| 0.7 μM | 1.2675 | 0.12809 | -0.8525 | 0.79772 | 1.2475 | 0.116 |
| 2 μM | 2.0625 | 0.40291 | -7.3025 | 0.60636 | 1.5875 | 0.27201 |
| 7 μM | 3.345 | 0.85018 | -15.245 | 1.11972 | 1.86 | 0.41172 |
| 20 μM | 4.62 | 1.12887 | -23.9175 | 0.78083 | 1.995 | 0.49155 |
| **Effects of 3,4,5 F-NAL-Phe (n=5)** | | | | | | |
| Concentration | I/I_0_ Mean | I/I_0_ SEM | ΔV_0.5_ (mV) Mean | ΔV_0.5_ (mV) SEM | G_max_/G­_max0_ Mean | G_max_/G­_max0_ SEM |
| 0.2 μM | 1.28765 | 0.26808 | 1.78 | 2.9335 | 1.41197 | 0.21357 |
| 0.7 μM | 3.01325 | 0.44971 | -8.28 | 2.11339 | 1.94982 | 0.29595 |
| 2 μM | 4.6518 | 0.70701 | -14.84 | 3.58951 | 2.24014 | 0.35646 |
| 7 μM | 6.60122 | 0.97463 | -26.44 | 4.16444 | 2.4095 | 0.37576 |
| 20 μM | 7.10772 | 0.99927 | -32.36 | 4.91839 | 2.35204 | 0.35225 |
| Table containing source data for the application of the PUFA analogues NALT, NAL-Phe, 4BR-NAL-Phe, 4F-NAL-PHe, and 3,4,5 F-NAL-Phe on the cardiac Kv7.1/KCNE1 at every concentration (0.2, 0.7, 2, 7, and 20 μM). | | | | | | |
