# Supplementary material for: Mechanistic insights into robust cardiac IKs potassium channel activation by aromatic polyunsaturated fatty acid analogues
Source: eLife. 2023 Jun 23;12:e85773. doi: 10.7554/eLife.85773 (PMC10328494; doi:10.7554/eLife.85773)
Supplement: Figure 4—source data 1. [file elife-85773-fig4-data1.docx]

| **Effects of NALT and 3F-NALT on IKs Channel** | | | | | | |
| --- | --- | --- | --- | --- | --- | --- |
| **Effects of NALT (n=4)** | | | | | | |
| Concentration | I/I_0_ Mean | I/I_0_ SEM | ΔV_0.5_ (mV) Mean | ΔV_0.5_ (mV) SEM | G_max_/G­_max0_ Mean | G_max_/G­_max0_ SEM |
| 0.2 μM | 0.94553 | 0.12581 | 3.575 | 0.38379 | 0.98609 | 0.03552 |
| 0.7 μM | 1.06501 | 0.1958 | 4.4 | 0.55827 | 1.13484 | 0.1989 |
| 2 μM | 1.9349 | 0.31466 | -4.95 | 2.75605 | 1.43115 | 0.25814 |
| 7 μM | 3.7006 | 0.66965 | -20.075 | 2.73538 | 1.53136 | 0.32306 |
| 20 μM | 5.14129 | 1.22612 | -56.125 | 3.58733 | 1.43185 | 0.31044 |
| **Effects of 3F-NALT (n=3)** | | | | | | |
| Concentration | I/I_0_ Mean | I/I_0_ SEM | ΔV_0.5_ (mV) Mean | ΔV_0.5_ (mV) SEM | G_max_/G­_max0_ Mean | G_max_/G­_max0_ SEM |
| 0.2 μM | 1.09839 | 0.09409 | -4.4965 | 0.54233 | 0.96486 | 0.19748 |
| 0.7 μM | 1.35173 | 0.22412 | -6.5088 | 1.40767 | 1.15049 | 0.18786 |
| 2 μM | 2.40013 | 0.49387 | -14.547 | 2.48184 | 1.37244 | 0.25805 |
| 7 μM | 5.3983 | 1.60062 | -40.014 | 2.89261 | 1.31846 | 0.26822 |
| 20 μM | 4.98494 | 1.02666 | -69.349 | 1.35624 | 1.27204 | 0.2935 |
| Table containing source data for the application of the PUFA analogues NALT and 3F-NALT on the cardiac Kv7.1/KCNE1 at every concentration (0.2, 0.7, 2, 7, and 20 μM). | | | | | | |
