# Supplementary material for: Mechanistic insights into robust cardiac IKs potassium channel activation by aromatic polyunsaturated fatty acid analogues
Source: eLife. 2023 Jun 23;12:e85773. doi: 10.7554/eLife.85773 (PMC10328494; doi:10.7554/eLife.85773)
Supplement: Figure 5—source data 1. [file elife-85773-fig5-data1.docx]

| **Effects of Lin-Tyr and 3,4,5 F NAL-Phe on WT and Mutated IKs Channels** | | | |
| --- | --- | --- | --- |
| **Effects of Lin-Tyr on WT IKs Channel (n=4)** | | | |
| Concentration | ΔV_0.5_ (mV) Mean | ΔV_0.5_ (mV) SEM |  |
| 0.2 μM | 0.21253 | 2.22787 |  |
| 0.7 μM | -8.4223 | 1.76905 |  |
| 2 μM | -23.405 | 1.6272 |  |
| 7 μM | -55.606 | 1.95738 |  |
| 20 μM | -74.426 | 4.13763 |  |
| **Effects of Lin-Tyr on R231Q+Q234R IKs Channel (n=4)** | | | |
| Concentration | ΔV_0.5_ (mV) Mean | ΔV_0.5_ (mV) SEM |  |
| 0.2 μM | 5.518 | 3.28649 |  |
| 0.7 μM | -3.096 | 6.25581 |  |
| 2 μM | -12.104 | 5.38694 |  |
| 7 μM | -36.534 | 6.2442 |  |
| 20 μM | -36.536 | 7.31673 |  |
| **Effects of 3,4,5 F-NAL-Phe on WT IKs Channel (n=5)** | | | |
| Concentration | G_max_/G­_max0_ Mean | G_max_/G­_max0_ SEM |  |
| 0.2 μM | 1.41197 | 0.21357 |  |
| 0.7 μM | 1.94982 | 0.29595 |  |
| 2 μM | 2.24014 | 0.35646 |  |
| 7 μM | 2.4095 | 0.37576 |  |
| 20 μM | 2.35204 | 0.35225 |  |
| **Effects of 3,4,5 F-NAL-Phe on K326C IKs Channel (n=3)** | | | |
| Concentration | G_max_/G­_max0_ Mean | G_max_/G­_max0_ SEM |  |
| 0.2 μM | 1.08667 | 0.06667 |  |
| 0.7 μM | 1.22333 | 0.11865 |  |
| 2 μM | 1.29 | 0.14189 |  |
| 7 μM | 1.29 | 0.15535 |  |
| 20 μM | 1.22 | 0.15308 |  |
| Table containing source data for the application of the PUFA analogues Lin-Tyr on the WT cardiac Kv7.1/KCNE1 and with the mutation R231Q+Q234R and 3,4,5 F-NAL-Phe on the cardiac Kv7.1/KCNE1 and with the mutation K326C at every concentration (0.2, 0.7, 2, 7, and 20 μM). | | | |
