# Supplementary material for: Mechanistic insights into robust cardiac IKs potassium channel activation by aromatic polyunsaturated fatty acid analogues
Source: eLife. 2023 Jun 23;12:e85773. doi: 10.7554/eLife.85773 (PMC10328494; doi:10.7554/eLife.85773)
Supplement: Figure 6—source data 1. [file elife-85773-fig6-data1.docx]

| **Effects of NALT and NAL-Phe on IKs Channel Mutations removing hydrogen bonding residues in the S3-S4 loop** | | | |  |
| --- | --- | --- | --- | --- |
| **Effects of NALT on WT IKs (n=4)** | | | |  |
| Concentration | ΔV_0.5_ (mV) Mean | ΔV_0.5_ (mV) SEM |  |  |
| 0.2 μM | 3.575 | 0.38379 |  |  |
| 0.7 μM | 4.4 | 0.55827 |  |  |
| 2 μM | -4.95 | 2.75605 |  |  |
| 7 μM | -20.075 | 2.73538 |  |  |
| 20 μM | -56.125 | 3.58733 |  |  |
| **Effects of NAL-Phe on WT IKs (n=4)** | | | |  |
| Concentration | ΔV_0.5_ (mV) Mean | ΔV_0.5_ (mV) SEM |  |  |
| 0.2 μM | 1.23454 | 0.35356 |  |  |
| 0.7 μM | 0.90146 | 0.20057 |  |  |
| 2 μM | -1.6853 | 1.21466 |  |  |
| 7 μM | -7.5297 | 3.02791 |  |  |
| 20 μM | -12.527 | 3.76753 |  |  |
| **Effects of NALT on S217A IKs (n=5)** | | | |  |
| Concentration | ΔV_0.5_ (mV) Mean | ΔV_0.5_ (mV) SEM |  |  |
| 0.2 μM | -0.57916 | 0.345061 |  |  |
| 0.7 μM | -4.15906 | 0.546143 |  |  |
| 2 μM | -11.4304 | 1.576021 |  |  |
| 7 μM | -34.8116 | 2.07221 |  |  |
| 20 μM | -65.9443 | 3.749217 |  |  |
| **Effects of NALT on Q220L IKs (n=3)** | | | |  |
| Concentration | ΔV_0.5_ (mV) Mean | ΔV_0.5_ (mV) SEM |  |  |
| 0.2 μM | -1.52122 | 1.515092 |  |  |
| 0.7 μM | -4.35308 | 0.736832 |  |  |
| 2 μM | -8.23543 | 5.241416 |  |  |
| 7 μM | -31.6237 | 6.375108 |  |  |
| 20 μM | -59.5385 | 11.10041 |  |  |
| **Effects of NALT on T224V IKs (n=4)** | | | | |
| Concentration | ΔV_0.5_ (mV) Mean | ΔV_0.5_ (mV) SEM |  |  |
| 0.2 μM | 2.591954 | 3.26478 |  |  |
| 0.7 μM | 0.98816 | 4.440905 |  |  |
| 2 μM | 1.9982 | 2.919261 |  |  |
| 7 μM | -13.5565 | 4.524764 |  |  |
| 20 μM | -32.1606 | 6.970673 |  |  |
| **Effects of NALT on S225A IKs (n=7)** | | | | |
| Concentration | ΔV_0.5_ (mV) Mean | ΔV_0.5_ (mV) SEM |  |  |
| 0.2 μM | 0.485872 | 1.303365 |  |  |
| 0.7 μM | -2.1039 | 1.703382 |  |  |
| 2 μM | -6.20795 | 2.586843 |  |  |
| 7 μM | -32.5378 | 3.063233 |  |  |
| 20 μM | -52.3833 | 3.742495 |  |  |
| **Effects of NAL-Phe on T224V IKs (n=7)** | | | | |
| Concentration | ΔV_0.5_ (mV) Mean | ΔV_0.5_ (mV) SEM |  |  |
| 0.2 μM | -3.38759 | 2.55998 |  |  |
| 0.7 μM | -1.93549 | 2.00388 |  |  |
| 2 μM | -1.8096 | 2.10132 |  |  |
| 7 μM | -4.62585 | 1.98088 |  |  |
| 20 μM | -13.1987 | 2.57089 |  |  |
| Table containing source data for the application of the PUFA analogues NALT on the WT cardiac Kv7.1/KCNE1 and with mutations S217A, Q220L,T224V and S225A and NAL-Phe on WT cardiac Kv7.1/KCNE1 and with the mutation T224V at every concentration (0.2, 0.7, 2, 7, and 20 μM). | | | |  |
